# A Self-Propelled Linear Piezoelectric Micro-Actuator Inspired by the Movement Patterns of Aquatic Beetles

**DOI:** 10.3390/mi15101197

**Published:** 2024-09-27

**Authors:** Xinjie Wang, Gen Wang

**Affiliations:** School of Mechanical Engineering, Nanjing University of Science and Technology, Nanjing 210094, China; genwang36@njust.edu.cn

**Keywords:** self-propelled piezoelectric linear micro-actuator, resonant type, driving characteristics, finite element simulation

## Abstract

The locomotion mechanisms and structural characteristics of insects in nature offer new perspectives and solutions for designing miniature actuators. Inspired by the underwater movement of aquatic beetles, this paper presents a bidirectional self-propelled linear piezoelectric micro-actuator (SLPMA), whose maximum size in three dimensions is currently recognized as the smallest known of the self-propelled piezoelectric linear micro-actuators. Through the superposition of two bending vibration modes, the proposed actuator generates an elliptical motion trajectory at its driving feet. The size was determined as 15 mm × 12.8 mm × 5 mm after finite element analysis (FEA) through modal and transient simulations. A mathematical model was established to analyze and validate the feasibility of the proposed design. Finally, a prototype was fabricated, and an experimental platform was constructed to test the driving characteristics of the SLPMA. The experimental results showed that the maximum no-load velocity and maximum carrying load of the prototype in the forward motion were 17.3 mm/s and 14.8 mN, respectively, while those in the backward motion were 20.5 mm/s and 15.9 mN, respectively.

## 1. Introduction

Piezoelectric actuators, with the rise of smart materials and the progress of modern control technology, have developed rapidly in recent decades and have gradually become highly competitive for micro-actuation [1,2,3]. Piezoelectric actuators exhibit characteristics such as strong resistance to magnetic interference, fast response speed, self-locking, and compact structure [4,5,6,7]. Due to their excellent performance, they have been widely applied in fields such as aerospace, precision equipment, and medical devices [8,9,10]. The core principle of these actuators is to convert electrical energy from piezoelectric materials into mechanical deformation, generating elliptical trajectories at certain driving points, thereby achieving macroscopic motion through frictional contact with the moving element [11,12,13]. There are several ways to classify piezoelectric actuators. Generally, based on the operating modes of PZT elements, linear piezoelectric actuators can be classified into sandwich-type and bonded-type actuators [14,15,16]. Compared to sandwich-type actuators, bonded-type actuators are typically smaller. They are better suited to miniaturized applications. According to their output modes, they can be further categorized into linear and rotary actuators [17,18,19,20]. Additionally, piezoelectric actuators can be divided into self-propelled and non-self-propelled types, depending on whether the piezoelectric vibrator simultaneously functions as both the mover and the stator [21]. In summary, the different operating principles of actuators provide greater possibilities for various applications.

Currently, research into rotary ultrasonic motors and non-self-propelled linear ultrasonic actuators is a significant focus of researchers. Studies have reported plenty of different structures and their corresponding actuating methods. For example, Bai et al. [22] designed a bolt-clamped piezoelectric actuator, which outputs rotational motion at the top and bottom of a 35 mm × 40 mm vibrator. With a preload of 11 N, the peak speed reaches 342 rpm, and, at a speed of 60 rpm, the maximum torque is 722 mN·m. Zhu et al. [23] proposed a novel hollow piezoelectric stack micro-rotary actuator with dimensions of 50 mm × 6 mm × 6 mm (with a 2 mm through hole), achieving a maximum speed of 913 rpm at a rotational speed of 450 rpm, with a maximum load of 70.3 mN. Izyhara et al. [24] developed a micro-linear piezoelectric ultrasonic motor with a thin hollow design, limited to 4.5 mm × 4.5 mm × 0.33 mm, which achieves a maximum thrust of 12.9 mN and a linear speed of 92.8 mm/s; in addition, it employs an elastic cylindrical slider to generate preload. Li et al. [25] proposed a novel linear piezoelectric micro-actuator with a miniaturized size of 7.4 mm × 7.4 mm × 10 mm, achieving maximum speeds during forward motion of 19.06 mm/s, a resolution of 0.3 μm, and a thrust of 17.45 mN; for reverse motion, the maximum speed, resolution, and thrust are 16.92 mm/s, 0.45 μm, and 15.28 mN, respectively. Tanoue et al. [26] introduced a new type of ultrasonic linear motor that features a quadrupole stator driving a slider, with dimensions of approximately 20 mm × 10 mm × 5 mm. The maximum speed is 258 mm/s, with a maximum thrust of 490 mN.

However, the performance of self-propelled ultrasonic actuators that have been reported to date is not entirely satisfactory. Some researchers have designed the motion rules of these actuators to resemble the movement of a caterpillar to enhance their driving characteristics, incorporating additional PZT elements, which increases the overall structural complexity. For example, Deng et al. [27] introduced a compact resonant caterpillar-like piezoelectric robot characterized by six driving feet evenly arranged around its circumference, resembling a dumbbell structure. The overall size of this robot is 30 mm × 30 mm × 65 mm, with a maximum speed of 200 mm/s and a displacement resolution of 0.71 μm. Sangchap et al. [28] designed a uniaxial walking caterpillar actuator with dimensions of approximately 55 mm × 23.1 mm × 15 mm, achieving a resolution and speed of 81 nm and 1.075 mm/s, respectively. Other scholars have chosen to explore piezoelectric resonant self-propelled actuators, but they remain somewhat large. Bai et al. [29] designed a bidirectional self-propelled linear piezoelectric actuator with dimensions of 28 mm × 5 mm × 5 mm, achieving no-load speeds of 43.76 mm/s and 43.14 mm/s. Zhang et al. [30] proposed a frog-shaped linear piezoelectric actuator with a sandwich structure that generates linear motion only by first-order longitudinal vibration. Its dimensions are 107 mm × 56.96 mm × 15 mm, and the actuator has a maximum no-load speed of 287 mm/s under an excitation voltage of 275 V and can provide a maximum thrust of 11.8 N under a preload of 60 N.

As mentioned above, it is not difficult to see that these self-propelled piezoelectric actuators still have room for development in terms of spatial size and the number of PZT elements. Therefore, this paper proposes a miniaturized self-propelled micro-actuator. Through a biomimetic structural design, the size of the PZT elements is effectively increased while reducing the overall dimensions, successfully fabricating a prototype of the actuator. This SLPMA is expected to provide new options for optical devices in medical applications [31].

## 2. Structure Design and Working Principle

### 2.1. Biomimetic Design of the SLPMA

**Figure 1:**
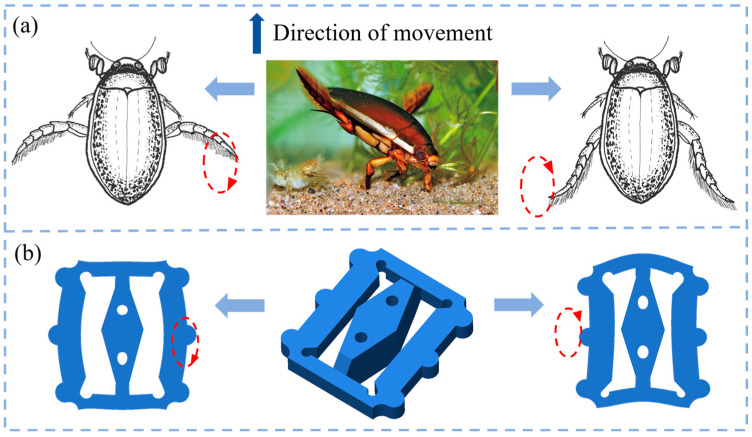
Biomimetic principles and motion mechanism. (**a**) Motion mechanism of aquatic beetle underwater; (**b**) fundamental principle of vibrator motion.

**Figure 2:**
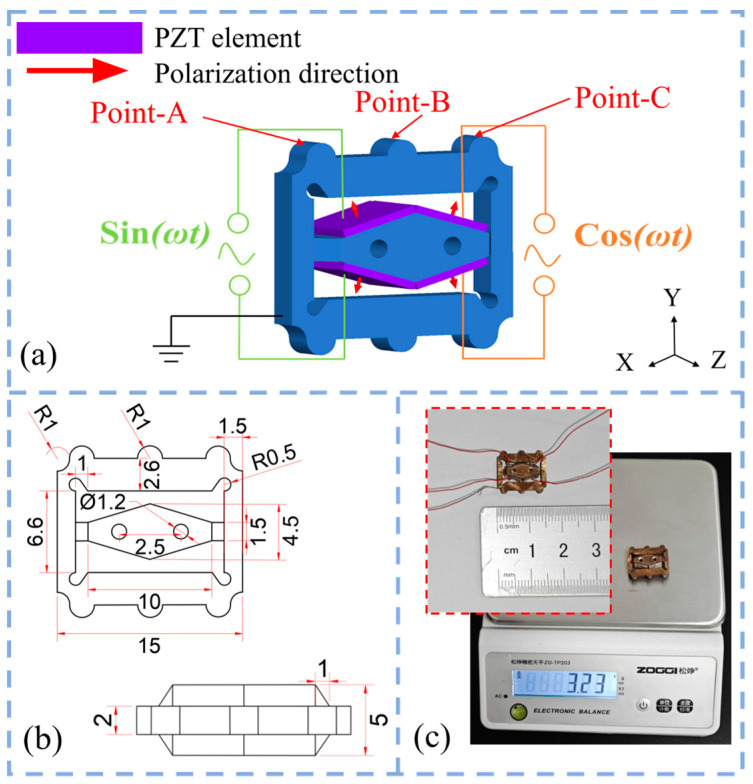
Self-propelled piezoelectric linear micro-actuator. (**a**) Voltage actuation for the PZT elements; (**b**) detailed dimensions of the micro-actuator; (**c**) micro-actuator prototype.

The predatory aquatic beetle is a commonly observed species in underwater ecosystems. As illustrated in Figure 1a, the forelimbs of the aquatic beetle have evolved specifically to capture and grasp prey, while the hind limbs are adapted for propulsion through the water. The insect can move forward because of reciprocal circular motions performed with its two hind limbs. Based on this biological method, the fundamental structure of a metal vibrator was designed, as shown in Figure 1b. There are two kinds of protrusions on the actuator. Middle protrusions, named ‘point-B’ in Figure 2a, are on two sides of the structure. They are actuated as propulsion mechanisms to move in ellipses. The protrusions at the top corners are named ‘points-A’ and ‘point-C’, which act as guides. When combining the two kinds of protrusions, the vibrator’s motion can be similar to the motion of a swimming aquatic beetle. The following section provides a detailed discussion of the working mechanism of the SLPMA and the configuration of the PZT elements.

### 2.2. Working Principle of the SLPMA

Figure 2 illustrates the self-propelled piezoelectric linear micro-actuator, which consists of an external square frame and an internal diamond-shaped excitation structure. Each corner on the square frame features guide feet with a semicircular shape, which ensure that the vibrator remains parallel to the track wall. The central semicircular protrusions are called driving feet, which provide the driving force through frictional contact with the track wall. Since the thickness of the micro-actuator is only 2 mm, it was necessary to attach PZT elements of 5 mm × 5 mm × 0.5 mm. Therefore, the thickness of the internal rhombic excitation structure is designed to be 5 mm. To effectively increase the amplitude and reduce the overall size, the edge length is more than 5 mm, and holes are drilled on the rhombus structure symmetrically (specific structure parameters are shown in Figure 2b). Additionally, a chamfering technique was employed to connect the terminal ends of the rhombus to the square frame. Figure 2a illustrates the placement of the PZT elements and their electrical connections. The red arrows indicate the polarization direction of the PZT elements, which are polarized in the d_31_ direction. A sinusoidal voltage signal is applied to the PZT elements, with two channels having the same frequency and a phase difference of 90°. This design allows the driving feet to move elliptically using only four piezoelectric ceramic pieces. The symmetrical design ensures the synchronization of the driving feet on both sides.

**Figure 3:**
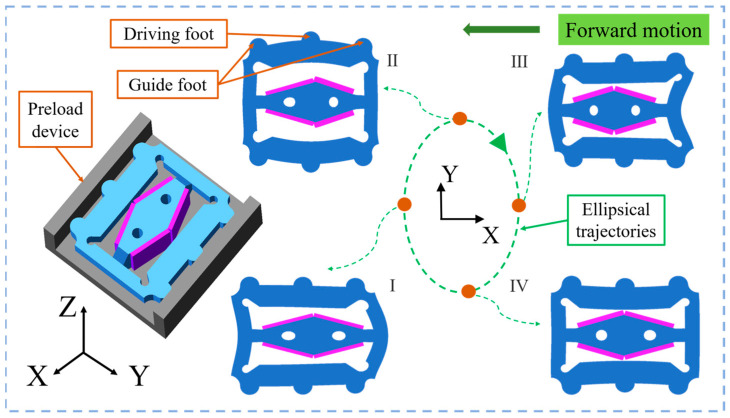
The motion process of the micro-actuator within one cycle.

Figure 3 illustrates the complete motion of the micro-actuator over one cycle. It is worth noting that the preloading device was simplified to illustrate how the micro-actuator was positioned within the track. The voltage configuration methodology is implemented as illustrated in Figure 2a. Under the action of sine and cosine voltage signals, the driving feet of the vibrator produce an elliptical trajectory that generates an ellipse that rotates clockwise. The micro-actuator operates in the sequence (I), (II), (III), (IV), and (I). Ultimately, this actuator produces a macroscopic forward motion when an appropriate preload is applied. Furthermore, if the voltage signals reverse, the micro-actuator will move in the opposite direction.

## 3. Simulation Validation and Mathematical Analysis

### 3.1. Modal Analysis

To ascertain the structural parameters of the micro-actuator and to validate the feasibility of the driving principle, a modal analysis of the vibrator was conducted utilizing finite element analysis (FEA). The material selected for the vibrator was phosphor bronze, characterized by a density of 8800 kg/m^3^, a Young’s modulus of 113 GPa, and a Poisson’s ratio of 0.33. The PZT element employed in this study was a PZT-8, which possesses a density of 7600 kg/m^3^ and a Poisson’s ratio of 0.31. The specific material parameters are detailed in Table 1. The boundary conditions for the simulations were established as free, with the interface between the PZT elements and the vibrator as bonded contact in the FEA. The effects of epoxy resin were not considered in this analysis.

**Figure 4:**
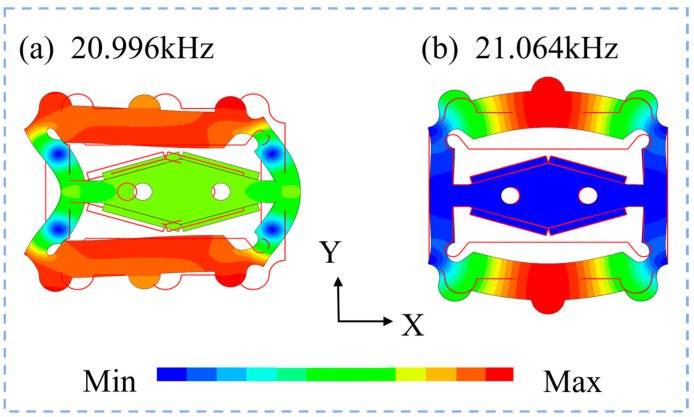
Mode deformation and frequency.

Through structural dimension optimization, the difference between the two modal frequencies was minimized as much as possible to ensure that the maximum amplitude was generated by applying voltages of the same frequency with different phases. Figure 4 shows the bending vibration mode frequencies and deformation quantities for both modes from the 3D simulation results. The working frequencies of the vibration mode were 20.996 kHz and 21.064 kHz, resulting in a frequency difference of only 68 Hz.

### 3.2. Transient Dynamics Analysis

**Figure 5:**
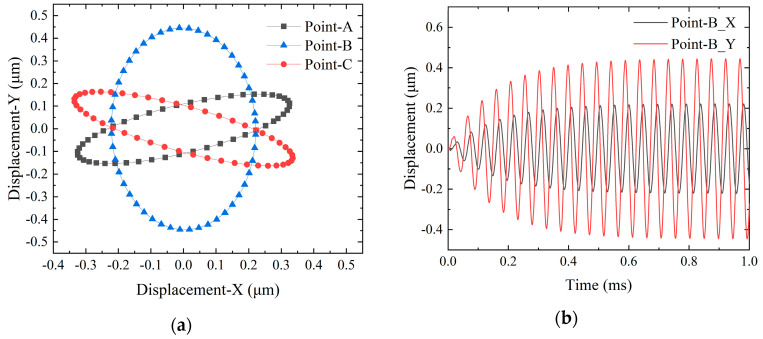
Simulation data results: (**a**) X-Y displacement trajectory simulation curves. (**b**) Displacement-time curve of point-B.

According to modal analysis, the vibrator is capable of generating an elliptical trajectory at the driving feet. To further explore the coupling phenomena between the two bending vibration modes, a transient analysis of the SLPMA was performed using FEA. This analysis utilized a damping ratio of 0.5%, with the boundary conditions set to free and the interface between the PZT elements and the vibrator as bonded contact. The selected excitation frequency was 21,030 Hz, representing the midpoint value between the two operational frequencies. A peak-to-peak voltage of 160 V_p-p_ was applied, and the configuration for the two-phase voltage application is illustrated in Figure 2a. The X-Y motion trajectory curves for the vertices of point-A, -B, and -C on one side of the micro-actuators’ driving and guiding feet are plotted in Figure 5a, and it illustrates the relationship between the time–displacement curves for point-B in Figure 5b. The elliptic trajectory curve corresponding to point-B can be fitted to yield the standard equation of an ellipse:

(1) $$\frac{x^{2}}{{\left(0.22\right)}^{2}}+\frac{y^{2}}{{\left(0.44\right)}^{2}}=1$$

The trajectory of the driving feet of the vibrator is verified by a mathematical model in the Appendix A.

## 4. Experimental Measurement

### 4.1. Impedance Testing

Figure 2c shows the assembled prototype, measuring 15 mm × 12.8 mm × 5 mm and weighing only 3.65 g. The PZT elements were bonded to both sides of the vibrator using epoxy resin, and, after the adhesive fully cured for 24 h, silver-plated wires of 0.3 mm were soldered onto the surface of the PZT elements, with the metal vibrator grounded. Based on simulation data and operational principles, the main factors affecting the micro-actuator’s movement included the peak-to-peak voltage and frequency of the excitation signal. Therefore, excitation voltage signals with different frequencies and peak values were used to test the driving characteristics of the micro-actuator.

**Figure 6:**
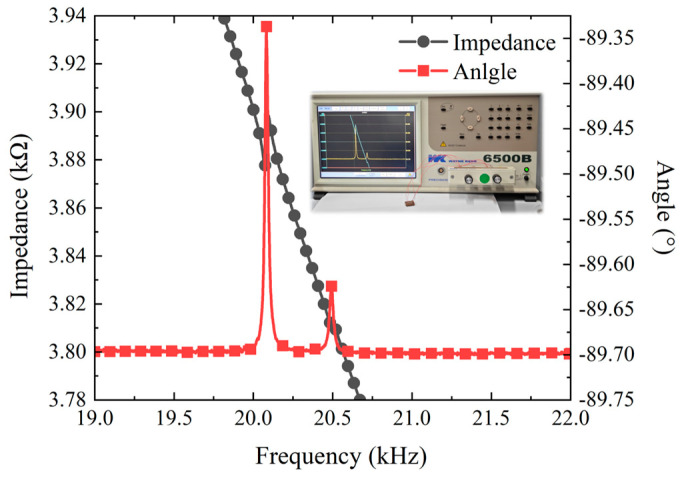
Impedance-phase curve.

Before testing, the impedance characteristics of the actuator were measured to determine the working frequency range. As shown in Figure 6, the positive and negative terminals of the micro-actuator were connected to the ports of the impedance analyzer, and the frequency swept from 19 to 23 kHz. The results indicated that the two working frequencies of the SLPMA were approximately 20.1 kHz and 20.5 kHz, with a frequency difference of about 400 Hz. The deviation between the analysis result and the measuring result may have been caused by manufacturing imperfections and inconsistent PZT elements bonding, yet it was still within tolerable limits.

### 4.2. Vibration Modal Testing

**Figure 7:**
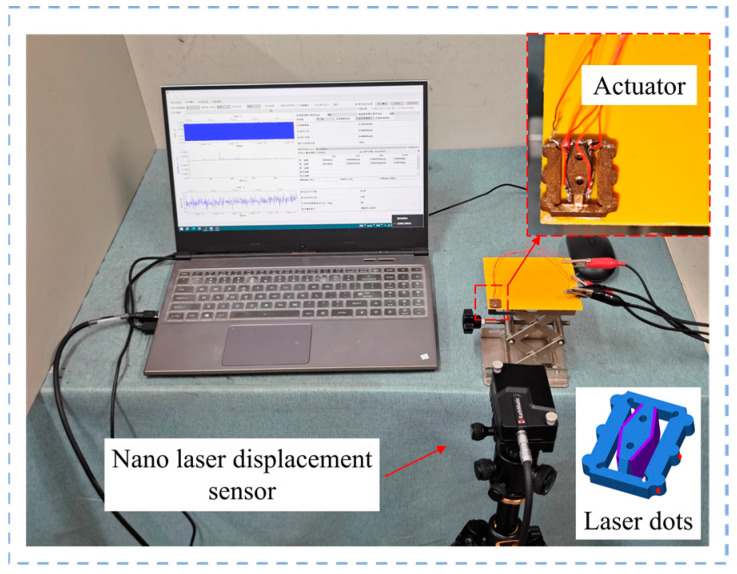
Modal amplitude testing experimental setup.

**Figure 8:**
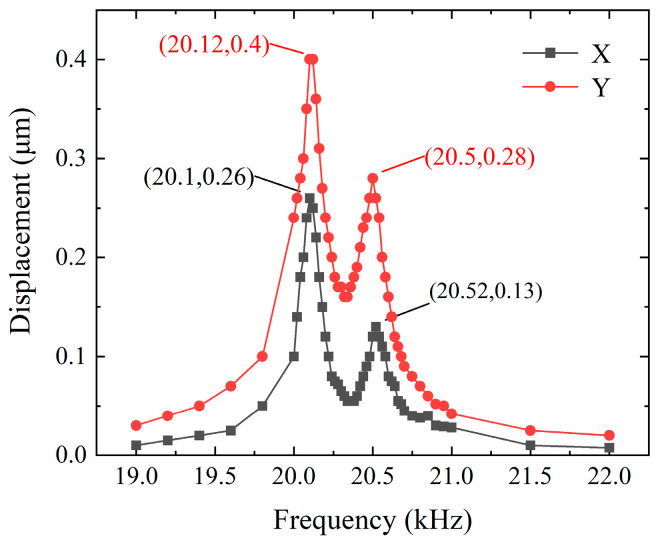
The displacements of driving feet versus frequency.

Figure 7 shows the experimental setup for measuring the driving feet amplitude and working frequency of the self-propelled micro-actuator. Firstly, the excitation voltage applied to the micro-actuator was configured according to Figure 2a, setting the output parameters of the signal generator so that the output voltage signal had a phase difference of 90° with a peak-to-peak value of 1 V. The frequency range swept from 19 kHz to 20 kHz. Then, two channels of excitation voltage signals were connected to a power amplifier to amplify the voltage 160 times before applying it to the actuator. At this point, a nano-laser sensor was aimed at the end of the driving feet to measure the *Y*-axis vibration. Since the amplitude in the *X*-axis direction of the driving feet (i.e., point-B) was difficult to measure, the laser point was aligned with one side of the guiding feet (i.e., point-A) for measurement, as shown in Figure 7. The displacements of the driving feet are illustrated in Figure 8. Measurements at two points revealed noticeable peaks at both 20.5 kHz and 20.1 kHz, with the maximum no-load amplitudes occurring at a frequency of 20.1 kHz, reaching 0.4 μm and 0.26 μm. At a frequency of 20.5 kHz, the displacements were 0.28 μm and 0.13 μm. These results are close to the simulation data.

### 4.3. Driver Characterization Testing

**Figure 9:**
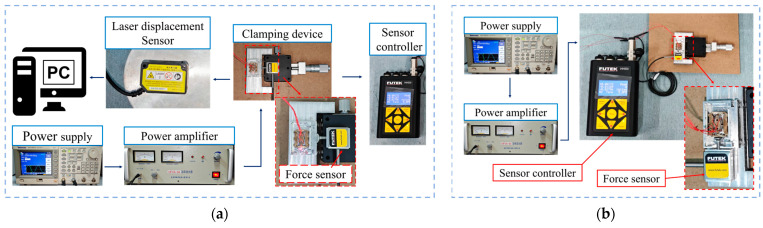
Driving characteristic testing setup. (**a**) Velocity testing; (**b**) output force testing.

Before testing the driving characteristics of the SLPMA, a preload device needed to be installed. As shown in Figure 9a, the preload device consisted of a micro-positioning platform, a soft spring, and a custom-made track. The preload was applied to one side of the movable baffle on the custom track by controlling the spring through the micro-positioning platform, thus providing the micro-actuator with an appropriate preload. It is worth noting that due to the machining accuracy problem, the surface machining of the aluminum alloy track had stripes (visible to the naked eye), resulting in insufficient contact friction, so ceramic sheets were pasted on both sides of the track to increase the friction.

**Figure 10:**
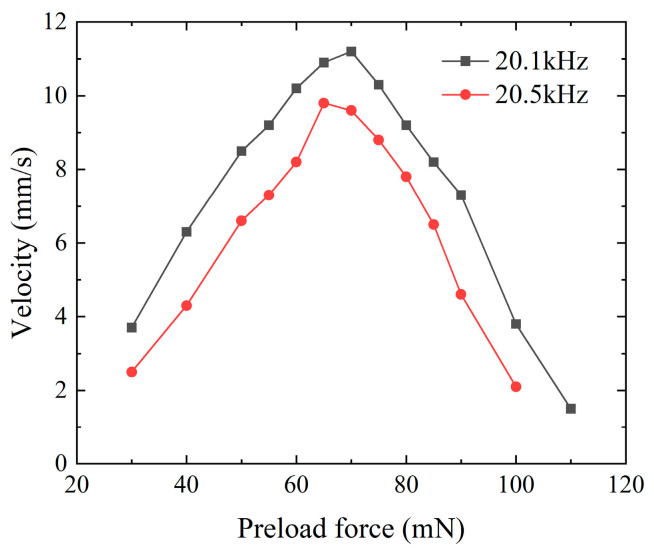
The velocities of the piezoelectric actuator with the different preloads.

The voltage excitation signal for the micro-actuator was configured according to the connection method mentioned in Section 4.2. The laser sensor’s spot was strategically positioned at the midpoint of the micro-actuator, enabling computer-controlled real-time monitoring of its movement. Concurrently, the screw head at the sensor’s end was affixed to a baffle adjacent to the track to measure the preload applied to the system accurately. Since the preload value influenced the performance of the actuator, the optimum preload force was investigated first. According to Figure 8, if the actuator was applied at 160 V_p-p_ at frequencies 20.1 kHz and 20.5 kHz, the velocities of the actuator with the different preloads were different. As shown in Figure 10, the optimum preload force was 70 mN.

The setup for the driving force testing platform is illustrated in Figure 9b. By attaching a force sensor to one end of the track, the actuator collided with the screw head; the output force was transmitted through the screw head and was displayed as a specific value on the measuring instrument. The preload was adjusted, and the final results were recorded when the data stabilized and reached the maximum value. The voltage excitation signal amplitude for the micro-actuator’s forward motion ranged from 120 V to 280 V, with data collected every 40 V. The frequency signal was measured between 20.4 kHz and 20.7 kHz, with data taken every 50 Hz. For backward motion, frequencies from 20 kHz to 20.4 kHz were measured while swapping the two-phase voltage excitation signals applied to the PZT elements, keeping other parameters consistent.

**Figure 11:**
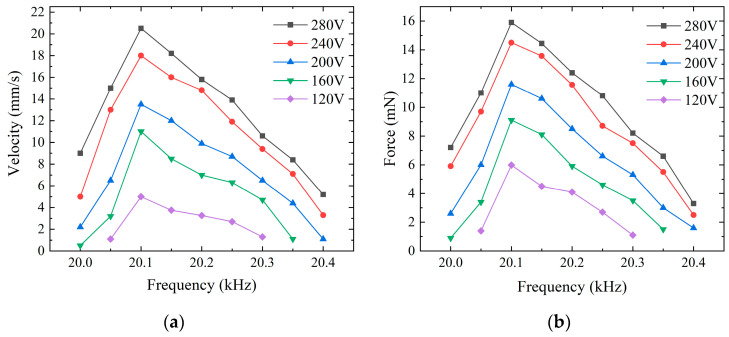
Forward motion characteristics at different voltages. (**a**) Relationship between frequency and speed; (**b**) relationship between frequency and output force.

**Figure 12:**
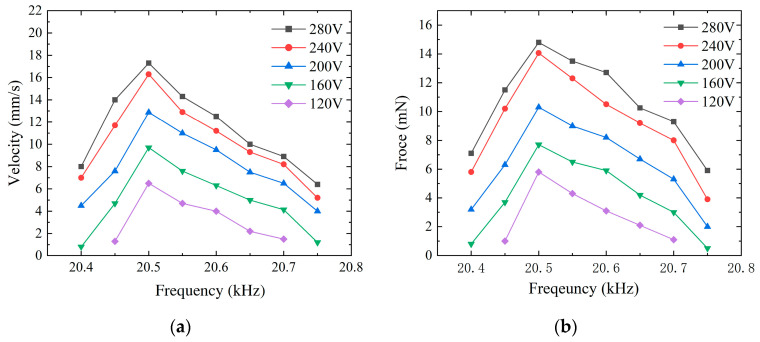
Backward motion characteristics at different voltages. (**a**) Relationship between frequency and speed; (**b**) relationship between frequency and output force.

The experimental results indicated that the operating frequencies for the maximum forward and backward speeds were not consistent. Despite some variance between the simulated and experimental data, the impedance and amplitude measurement results of the two were remarkably similar. According to Figure 11 and Figure 12, for the forward motion, the speed of the micro-actuator dropped to zero when the frequency surpassed the range of 20 kHz to 20.4 kHz; for the backward motion, the micro-actuator’s speed was null outside the frequency range of 20.4 kHz to 20.8 kHz. With voltage excitation at a frequency of 20.1 kHz, a peak-to-peak value of 280 V, and an optimum preload force of 70 mN, the maximum forward speed is 20.5 mm/s, and the maximum driving force is 15.9 mN. At a frequency of 20.5 kHz and the same peak-to-peak voltage and same preload force, the maximum backward speed is 17.3 mm/s, and the maximum driving force is 14.8 mN. There is a significant increase in forward driving force and speed with increasing voltage. However, as the voltage approaches 280 V, the rates of increase in driving force and speed begin to diminish, which may indicate that the maximum voltage of the PZT elements has been reached.

Theoretically, due to structural symmetry, bidirectional motion should be achieved at the same frequency by exchanging the applied cosine and sine voltage signals on the PZT elements. However, in practice, achieving these two modes of motion requires different operating frequencies. This discrepancy may be attributed to manufacturing and adhesive errors in the PZT elements, as well as possible errors in the processing and assembly of the preload device during the experimental process, leading to non-uniform preload distribution at the baffle and the influence of wiring on the micro-actuator.

The characteristics of the proposed SLPMAs were compared with those of several other similar SLPMAs, as listed in Table 2, in which the parameters are size, speed weight, and so on. Although the SLPMA was only a prototype proposed to verify the method of utilizing a symmetrical structure to design a self-propelled linear micro-actuator, the prototype exhibited some good performance compared to several similar SLPMAs, and the several advantages of the proposed SUSM are as follows:

(1) The SLPM actuator consists of an external square frame and an internal diamond-shaped excitation structure, with driving and guiding feet on the frame. It has a compact structure, which is conducive to miniaturization; the size and weight are limited to 15 mm × 12.8 mm × 5 mm and 3.23 g, respectively.
(2) Due to the symmetrical structural design, the SUSM can output bidirectional linear motion from the same sine and cosine signals; in addition, the simple structure facilitates machining.
(3) Benefiting from the symmetrical structure, the number of PZT elements required for assembly is minimized, facilitating easy construction, and bidirectional motion can be realized by the same vibration modes, which is conducive to the consistency of the characteristics between forward and backward motions.

**Table 2:** Performance comparison with other similar SLPMAs.

Parameters	Deng et al. [27]	Sangchap et al. [28]	Sun et al. [21]	Zhang et al. [30]	The SLPMA
Size (mm^3^)	30 × 30× 65	55 × 23.1 × 15	20 × 18.8 × 10	107 × 56.96 × 15	15 × 12.8 × 5
Weight (g)	56.7	/	/	1550	3.23
Frequency (kHz)	21.5	0.150	88.5	29.2	20.1/20.5
Voltage (V_p-p_)	200	20	120	275	280
Maximum thrust force (mN)	/	/	/	10,000	15.9/14.8
Maximum speed (mm/s)	200	1.075	120.6/130.1	241.6	20.5/17.3
Number of PZT elements	10	/	6	/	4

## 5. Conclusions

In this work, a self-propelled bidirectional piezoelectric linear micro-actuator (SLPMA), which is the smallest self-propelled piezoelectric actuator currently available, has been designed, inspired by the way aquatic beetles move underwater. It consists of a square outer frame structure and an inner diamond-shaped excitation structure with driving and guiding feet on the frame. It requires only four PZT elements of 5 mm × 5 mm × 0.5 mm and sinusoidal and co-sinusoidal voltage signals to achieve motion. In addition to its compact size and straightforward structure, the vibrator exhibits excellent symmetry, enabling it to effectively generate substantial amplitudes. The superposition of the two bending vibration modes facilitates the consistency of the forward and backward motions. The feasibility of the proposed scheme was verified through the FEA and mathematical modeling. A prototype was further fabricated to test its performance characteristics.

The experimental results show that under the optimal preload force of 70 mN and a peak-to-peak voltage of 280 V_p-p_, the forward operating frequency is 20.1 kHz, and the backward operating frequency is 20.5 kHz; under these conditions, the maximum speed of the forward motion reaches 20.5 mm/s with a maximum driving force of 15.9 mN, while the maximum speed of the backward motion reaches 17.3 mm/s with a maximum driving force of 14.8 mN. As expected, the forward and backward motion characteristics show relatively good consistency. This consistency can be further improved by improving the machining and assembly accuracy of the vibrator. This actuator is expected to provide new technology in the field of micromechanics.

## Figures and Tables

**Table 1:** Parameters of PZT elements.

Stiffness Coefficient Matrix/1010 (N·m^2^)		Piezoelectric Constant Matrix/(C·m^−2^)		Relative Dielectric Coefficient Matrix	
c11	14.4	e15	11.7	ε11	770
c12	7.8	e24	11.7	ε33	550
c13	7.8	e31	−4.2		
c33	12.4	e33	14.0		
c44	3.0				
c66	3.3				

## Data Availability

All data are presented within the paper.

## Appendix A

Due to the operation of the piezoelectric ceramic in d31 mode, standing wave displacements are generated at the two side ends by compressing the rhombic structure. It is assumed that the displacement of the piezoelectric ceramic excited at the operating frequency is *U*. Given the symmetry of the model, it can be deduced that the amplitude of the output displacements at the two ends is approximately equal without considering the interaction between the PZT elements. The following equation can express the relationship between displacement and time:

(A1) $$\left\{ \begin{array}{l} u_{R}=U\cos2\pi ft\\ u_{L}=U\sin2\pi ft \end{array}\right.$$

where *f* represents the vibration frequency, and *t* represents the vibration time.

When calculating the X-Y direction displacement at point-B, the rods on both sides are simplified as a cantilever beam structure and treated as a single elastic system. In this case, a conversion factor *k* is introduced for the X-Y direction displacement, so the displacement of point-B in the X-Y direction can be expressed as

(A2) $$\left\{ \begin{array}{l} u_{y}=k_{1}\left(u_{R}-u_{L}\right)\\ u_{x}=k_{2}(u_{R}+u_{L})/2 \end{array}\right.$$

Combining Equations (A1) and (A2), one can obtain

(A3) $$\left\{ \begin{array}{l} u_{y}=\sqrt{2}kU\cos(2\pi ft+\pi /4)\\ u_{x}=\sqrt{2}U\sin(2\pi ft+\pi /4)/2 \end{array}\right.$$

Establish the following relationship:

(A4) $$\frac{u_{x}^{2}}{U^{2}/2}+\frac{u_{y}^{2}}{2k^{2}U^{2}}=1$$

From this equation, it can be seen that a standard elliptic equation is formed in the XOY plane, which is in general agreement with the equation of the fitted curve. It is shown that point-B can produce an elliptical driving trajectory with proper voltage application. Mathematical analysis confirms the feasibility of this structural design.

## References

[1] Mohith S., Upadhya A.R., Navin K.P., Kulkarni S.M., Rao M. (2020). Recent Trends in Piezoelectric Actuators for Precision Motion and Their Applications: A Review. Smart Mater. Struct..

[2] Jain T., Sunil B.D.Y., Hasan M.A., Jain A., B S., Chahuan N. (2024). Smart Materials for Sensing and Actuation: State-of-the-Art and Prospects. E3S Web Conf..

[3] Rajeev A., Yin L., Kalambate P.K., Khabbaz M.B., Trinh B., Kamkar M., Mekonnen T.H., Tang S., Zhao B. (2024). Nano-Enabled Smart and Functional Materials toward Human Well-Being and Sustainable Developments. Nanotechnology.

[4] Wang C., Li H., Zhang Z., Yu P., Yang L., Du J., Niu Y., Jiang J. (2022). Review of Bionic Crawling Micro-Robots. J. Intell. Robot. Syst..

[5] Wang C., Zhang W., Zhao J., Hu J., Zou Y. (2020). Design, Takeoff and Steering Torques Modulation of an 80-Mg Insect-Scale Flapping-Wing Robot. Micro Nano Lett..

[6] Ryndzionek R., Sienkiewicz Ł. (2021). A Review of Recent Advances in the Single- and Multi-Degree-of-Freedom Ultrasonic Piezoelectric Motors. Ultrasonics.

[7] Song S., Shao S., Xu M., Shao Y., Tian Z., Feng B. (2018). Piezoelectric Inchworm Rotary Actuator with High Driving Torque and Self-Locking Ability. Sens. Actuators A Phys..

[8] Jūrėnas V., Kazokaitis G., Vaškas D. Ultrasonic Motor with Spherical Rotor for Nanosatellite Orientation. Proceedings of the 2020 International Conference Mechatronic Systems and Materials (MSM).

[9] Holden J.P.F., Gan L., Sims-Williams D., Gilbert J., Osborne P., Bastankhah M. (2024). Characterisation and Integration of Piezoelectric Trimorph Actuators for Blade Active Surface Control on a Scaled Wind Turbine. J. Phys. Conf. Ser..

[10] Ahmad Fuaad M.R., Hasan M.N., Ahmad Asri M.I., Mohamed Ali M.S. (2023). Microactuators Technologies for Biomedical Applications. Microsyst. Technol..

[11] Behera B., Nemade H.B. (2017). Recent Developments of Piezoelectric Motors with Diverse Operating Principles. ISSS J. Micro Smart Syst..

[12] Ma D. Research Progress and Development Direction of Structural Optimization and Modeling of Traveling Wave Rotating Ultrasonic Motor. Proceedings of the 2021 4th World Conference on Mechanical Engineering and Intelligent Manufacturing (WCMEIM).

[13] Mukhopadhyay S., Behera B., Kumar J. (2021). A Brief Review on the Recent Evolution in Piezoelectric Linear Ultrasonic Motors. Eng. Res. Express.

[14] Yun H., Kong D., Aoyagi M. (2022). Development of a Multi-Drive-Mode Piezoelectric Linear Actuator with Parallel-Arrangement Dual Stator. Precis. Eng..

[15] Ghenna S., Bernard Y., Daniel L. (2023). Design and Experimental Analysis of a High Force Piezoelectric Linear Motor. Mechatronics.

[16] Dong H., Li T., Wang Z., Ning Y. (2020). Design and Experiment of a Piezoelectric Actuator Based on Inchworm Working Principle. Sens. Actuators A Phys..

[17] Kanada A., Mashimo T. (2020). Design and Experiments of Flexible Ultrasonic Motor Using a Coil Spring Slider. IEEE/ASME Trans. Mechatron..

[18] Yu P., Wang L., Zhang S., Jin J. (2022). Transfer Matrix Modeling and Experimental Verification of Forked Piezoelectric Actuators. Int. J. Mech. Sci..

[19] Lai Z., Chen B., Li J., Wang Y. A Compact Standing Wave Ultrasonic Motor Using Collinear Layout. Proceedings of the 2022 16th Symposium on Piezoelectricity, Acoustic Waves, and Device Applications (SPAWDA).

[20] Kazumi T., Kurashina Y., Takemura K. (2019). Ultrasonic Motor with Embedded Preload Mechanism. Sens. Actuators A Phys..

[21] Sun D., Tang Y., Wang J., Wang X. (2020). Design of an H-Shaped Linear Piezoelectric Motor for Safety and Arming Device. Sens. Actuators A Phys..

[22] Bai D., Quan Q., Tang D., Deng Z. (2019). Design and Experiments of a Novel Rotary Piezoelectric Actuator Using Longitudinal–Torsional Convertors. IEEE Access.

[23] Zhu B., Li C., Wu Z., Zhu X. (2023). Design and Dynamic Analysis of a Novel Compound Bending Hollow Piezoelectric Beam Miniature Rotary Actuator. Ultrasonics.

[24] Izuhara S., Mashimo T. (2021). Design and Characterization of a Thin Linear Ultrasonic Motor for Miniature Focus Systems. Sens. Actuators A Phys..

[25] Li J., Liu Y., Deng J., Zhang S., Chen W. (2022). Development of a Linear Piezoelectric Microactuator Inspired by the Hollowing Art. IEEE Trans. Ind. Electron..

[26] Tanoue Y., Morita T. (2020). Rod Drive Type Ultrasonic Linear Motor with Quadruped Stator. Jpn. J. Appl. Phys..

[27] Deng J., Yang C., Liu Y., Zhang S., Li J., Ma X., Xie H. (2023). Design and Experiments of a Small Resonant Inchworm Piezoelectric Robot. Sci. China Technol. Sci..

[28] Sangchap M., Tahmasebipour M., Tahmasebipour Y. (2022). A Linear Inchworm Piezomotor with a New Configuration: Design Considerations, Fabrication and Characterization. Iran. J. Sci. Technol. Trans. Mech. Eng..

[29] Bai K., Li C., Xi C. (2020). Design of Two-Way Self-Moving Linear Ultrasonic Motor. IOP Conf. Ser. Earth Environ. Sci..

[30] Zhang Q., Chen W., Liu Y., Liu J., Jiang Q. (2017). A Frog-Shaped Linear Piezoelectric Actuator Using First-Order Longitudinal Vibration Mode. IEEE Trans. Ind. Electron..

[31] Bhokare S.G., Behera B. (2022). Motion Improvised Miniaturized Dual Focus Lens Module Based on Piezoelectric Actuator for the Medical Applications. Ferroelectrics.

